# Reporting from the front

**DOI:** 10.1007/s11069-023-05815-3

**Published:** 2023-02-06

**Authors:** Christian Werthmann

**Affiliations:** Institute for Landscape Architecture, Leibniz University Hannover, Hanover, Germany

Reporting from the Front was the title of the 15th Venice Biennale in Architecture (2016) curated by the Pritzker Prize winning architect Alejandro Aravena. Aravena showcased architectural examples and approaches that tried to improve living conditions in tough urban situations on the planet. When I started writing this short communication, I felt the need to borrow his line, as this communication feels somewhat like a report from the frontline of urbanization.

In 2019, a multidisciplinary group of German researchers (landscape architecture, geoengineering, remote sensing, geoinformatics) and two small companies set out to develop an integrated early warning system (EWS) against landslides in the informally settled hills of Medellín, Colombia (funded by BMBF 03G0883A). Specifically, this was carried out in the settlement of Bello Oriente, a neighborhood located at the urban–rural border that has been affected by several landslides. The last one occurred in 2017 and destroyed six houses with no casualties. Now, we are approaching the end of a four-year living lab called *Inform@Risk* that involved 4,000 low-income residents, numerous city agencies, civil-based organizations and academics in Colombia.

The scope of *Inform@Risk* ranged from the abstractions of scientific work to implementation on the ground. The learnt lessons were plenty as we had to navigate the particular dynamics of self-constructed settlements, the changing personnel, politics and priorities of municipal government, a global shortage of chips and a pandemic. Our challenges and experiences relate very much to Jerold Kayden’s aphorism “To design is human, to implement, divine” (1). To build upon Rumsfeld’s infamous quote, our team tried to anticipate the known unknowns and hoped to master the unknown unknowns.

The somewhat easier solvable known unknowns were the physical aspects of the territory like the geophysical behavior of the mountain or the physical structure of the informal settlement. It was substantially harder to understand and navigate the social dynamics of the neighborhood which volunteered as a test case for the EWS. As no neighborhood is unequivocal, in our test community there also lived a multitude of actors with differing interests and leverage (family groups, ethnic groups, new arrivals, local NGO’s, land owners, gangs, etc.). From the outset, we expected a fair amount of social work and remediation. As low-income citizens of informal settlements are more vulnerable to shocks (sudden loss of income, changing government policies, increased settlement activity, destructive weather events, gang warfare, guerilla activity, etc.), there are also increased social dynamics in place. In a multi-year project, rapid change is to be expected, one just cannot predict its exact nature.

While there was continuous interest by many families and the leadership of the neighborhood to decrease their vulnerability against landslides, there was also a great fluctuation of involved inhabitants. Due to new arrivals (mostly refugees from Venezuela and displaced rural population from Colombia) or residents who left the area, or families, whose life circumstances suddenly changed, our team had to repeat the basic tenets of the effort time and again. This fluctuation is natural for low-income neighborhoods, but it was much more exacerbated through the pandemic (more about that later). The scope of our social engagement also widened as informal construction of houses continued in high hazard areas. The community workshops did not only focus on how to react in case of a landslide, but also how to prevent a landslide by not building in the wrong place.

Overall, the experiences of *Inform@Risk* definitely confirmed the wisdom that an EWS is foremost a social construct in need of continuous care and it cannot be merely seen as a combination of technical equipment. To secure this, continuation of social attention is most likely the biggest challenge, and it cannot solely rely on the responsible authorities. In order to be resilient, one has to ground it in the community itself, socially and physically, which brings me to the second known unknown: government support.

Political support is always a snapshot in time, it can undergo changes as political priorities and leadership can change quickly in unforeseen ways. One just does not know at the outset of a project, if, when, and in which direction change comes. In the beginning of the research project, all the relevant municipal authorities welcomed and vowed to support the three-year project. During the tenure of our project, we saw in the second year a new mayor, who exchanged leaders of agencies, who in turn exchanged staff. While the new mayor supports the project, we still had to explain the nature of it to every new person in charge from the beginning and garner his or her support. Being a project developed by a foreign partner country, the hand-over process entails administrative challenges to assure the continuation of the EWS once the project finishes, and even more, once the current government changes. In addition, a particular hardship proved to be the continuous weakening and still unsure future of Medellín’s premier monitoring and warning agency which is naturally the key partner for an EWS, but unfortunately got into the crossfire of politics.

As the particular value of a living lab is to learn from life, the navigation of changing politics is a natural part of it. As a researcher, one has to accept these hardships not as a nuisance, but as an opportunity to sharpen the research, especially the research questions. In our case, already lingering questions of self-sufficiency got more weight: can one design and implement an EWS that still functions on a minimal level with little government support?

Coming to the last part: the unknown unknowns. In our case, there are three: import and nationalization of goods, shortage of chips and Covid-19.

The first one is not a total unknown unknown. Bureaucracy and import issues can be tedious, but generally solvable. In our case, Colombian import and nationalization procedures are especially tough and cost us more time, money and efforts than expected, as there is not an international trade agreement between Colombia and Germany. In our case, we solved this issue by donating the instruments to a local civil society organization. Better would have been to have a powerful local research partner who could nationalize the instruments in its name. But, best would be to import as little as possible.

The second, the global shortage of chips starting in 2021, was a true unknown unknown for us as it was not on the horizon when we set up the project. Chips are part of the over 120 sensors to be installed in the hazardous slopes on our test site. Luckily our geoengineering and geoinformatics specialists developed makeshift solutions to work around the problem.

The last and most consequential unknown unknown hit us after the first year in the project: the Corona pandemic. For 18 months, our German team could not travel to Medellín, be there for longer periods, not hold in-person workshops with the community, not inspect the slopes or be physically present in the field. As a consequence, we transformed our travel budget into person hours and hired additional Colombian researchers to complement our team in Medellín. Our geoengineers developed with a great deal of effort detailed how-to-instructions for all their field work and managed to supervise the installation from a distance. The team started to set up online community workshops, where sometimes ten family members would huddle around the small screen of a smart phone. One resident volunteer started on his own initiative to go from house to house and explain landslide threat and the EWS test installation via posters that we had left on our last visit. Priorities shifted for many residents as they experienced a sharp drop of income through multiple lockdowns. With little to no savings, many inhabitants were naturally more worried about hunger than about landslides. In 2020, online meetings were a helpful crutch to not completely lose touch with the community, but it could not substitute the informational wealth and bonding experience of in-person congregations that are necessary to build up trust (the biggest currency in a project like this). Once community visits were again possible in spring 2021, our Colombian team members could commence in-person workshops. This was just in time as community engagement was at a low.

After getting a one-year extension on the project, we are now nearing the implementation of the monitoring instruments, getting ready to test and train the sensor system, develop and implement the warning dissemination and evacuation procedures with residents and municipal authorities and evaluate the information impact on the residents. Additional capacities inside the municipal authorities still have to be freed up to continue and improve the EWS once the project will be handed over. Over the course of the last three years, our municipal partners as they experienced a great deal of change and unrest, had always tried to master the described challenges, which leaves us confident to find solutions for the remaining unsolved issues.

As described in the beginning, the living lab *Inform@Risk* brought many challenges, but also invaluable lessons with it; if it is for the design of the EWS itself or regarding questions of replicability in other locations. While we will scientifically evaluate these lessons in the coming months, it already becomes clear that a truly sustainable and replicable EWS against landslides will have to have a safe-to-fail mode that still offers basic functions should technology collapse, political support end or finances dry up. This safe-to-fail mode will require the build-out of the self-help capacities of the residents to monitor their own territory, disseminate warnings and react to it without modern technology or government support.
